# Long-term effects of daylight saving time on driving fatigue

**DOI:** 10.1016/j.heliyon.2024.e34956

**Published:** 2024-07-20

**Authors:** Federico Orsini, Esther Dingena Domenie, Lisa Zarantonello, Rodolfo Costa, Sara Montagnese, Riccardo Rossi

**Affiliations:** a Department of Civil, Environmental and Architectural Engineering, University of Padua, Padua, Italy; b MoBe – Mobility and Behavior Research Center, University of Padua, Padua, Italy; c Department of General Psychology, University of Padua, Padua, Italy; d Department of Medicine, University of Padua, Padua, Italy; e Institute of Neuroscience, National Research Council (CNR), Padua, Italy; f Department of Biomedical Sciences, University of Padua, Padua, Italy; g Chronobiology Section, Faculty of Health and Medical Sciences, University of Surrey, Guildford, UK

**Keywords:** Daylight saving time, Driving fatigue, Driving simulator, Circadian rhythms, Misalignment, Sleep curtailment

## Abstract

The study of the relationship between Daylight Saving Time (DST) and road safety has yielded contrasting results, most likely in relation to the inability of crash-database approaches to unravel positive (ambient lighting-related) and negative (circadian/sleep-related) effects, and to significant geographical differences in lighting-related effects. The aim of this study was to investigate the effects of DST on driving fatigue, as measured by driving-based, physiological and subjective indicators obtained from a driving simulator experiment. Thirty-seven participants (73 % males, 23 ± 2 years) completed a series of 50-min trials in a monotonous highway environment: Trial 1 was in the week prior to the Spring DST transition, Trial 2 in the following week, and Trial 3 in the fourth week after the transition. Thirteen participants returned for Trial 4, in the week prior to the Autumn switch to civil time, and Trial 5 in the following week. Significant adverse effects of DST on vehicle lateral control and eyelid closure were documented in Trial 2 and Trial 3 compared to Trial 1, with no statistical differences between Trials 2 and 3. Further worsening in vehicle lateral control was documented in Trials 4 and 5. Eyelid closure worsened up to Trial 4, and improved in Trial 5. Participants were unaware of their worsening performance based on subjective indicators. In conclusion, DST has a detrimental impact on driving fatigue during the whole time during which it is in place. Such an impact is comparable, for example, to that associated with driving with a blood alcohol concentration of 0.5 g/L.

## Highlights

• DST had a negative short-term impact on objective driving fatigue indicators.
• Such negative effects persisted in the fourth week after the transition.
• Further worsening was observed at the very end of the DST period.
• Based on subjective indicators, participants were unaware of their worsening driving performance.

## Graphical abstract

**Figure:**
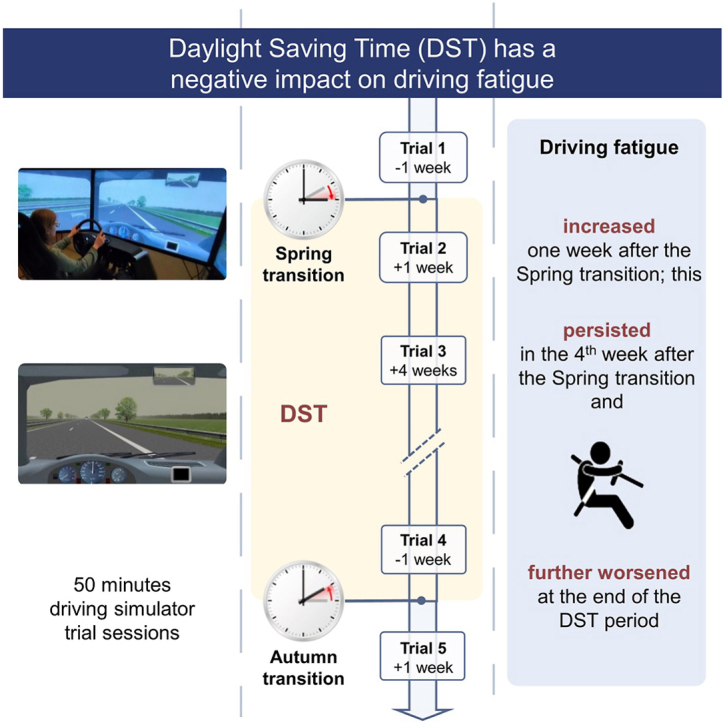

## 1 Introduction

As we move our clocks forward with the Spring transition to Daylight Saving Time (DST), we add 1 h to any degree of desynchrony between endogenous timing (dictated by our circadian clocks system, entrained to local solar time by light and dark cues), and the so-called social time (dictated by the existence of constraints such as time zones, work/study hours, and habits related to the availability of 24 h artificial lighting) [1]. Contrary to widespread belief, this extra hour desynchrony and its negative effects on sleep and daytime performance extend beyond the few days surrounding the Spring transition to DST, and most likely persists throughout the DST period [2,3].

The effect of DST on road safety has been investigated by several studies, with contrasting findings [[4], [5], [6], [7], [8], [9]]. Carey and Sarma [10] reviewed 24 studies published between 1974 and 2017, identifying inconsistent results both short- and long-term, and considering both the Spring transition to DST and the Autumn one from DST. This has been confirmed by more recent studies, some associating DST with overall positive outcomes [[11], [12], [13], [14], [15]], others with negative ones [[16], [17], [18], [19]], and others documenting no significant effects [20,21]. These divergent findings are most likely related to the dual impact of the Spring transition to DST. On one hand, this increases ambient lighting in the late afternoon, at a time when traffic volumes are typically high, while reducing it in the early morning, when traffic is generally lower (with local exceptions, and always in relation to geographical location). On the other hand, it negatively affects drivers, leading to circadian desynchrony and sleep curtailment, which in turn affect driving performance, with adverse consequences on road safety. These two effects are opposing and tend to counterbalance each other, as already highlighted by other authors [17,20]. Due to the reliance of most studies on historical crash data, it becomes complex to separate these two contributions, especially when considering the confounding effects of additional factors such as weather conditions or infrastructural features, which may also contribute to the overall outcome and possibly mask the impact of DST. Furthermore, the effect of DST on ambient lighting is strongly influenced by the specific location under investigation, including latitude, longitude, and time zone, thus also partly explaining the observed contrasting evidence. All the above issues also hinder our ability to accurately assess the magnitude of the effects of DST, which is crucial to correctly understand the phenomenon and identify adequate countermeasures.

These difficulties become even more pronounced when attempting to evaluate any long-term impact of DST, as seasonal factors also come into play, posing further challenges to statistical models. For instance, accounting for the seasonal variation in traffic flows alone may not suffice in certain regions, where the holiday season can influence traffic composition (e.g., fewer heavy-duty trucks and more private cars) and driver features (e.g., fewer commuters and more tourists), thus introducing additional opposing effects that become complex to model.

In one of our previous studies, we proposed an alternative approach to evaluate the sleep/circadian-related effects of DST on road safety, by use of a driving simulator [22]. We compared the driving performance of 23 young males before and after the transition to DST, and documented a negative effect on several driving safety-related indices. These included increased overtaking violations, longer reaction times, reduced proximity when overtaking a cyclist, and more abrupt exit maneuvers from a freeway [22].

We subsequently employed a similar driving simulator-based approach and experimental design to investigate, in a pilot study, the short-term sleep/circadian effects of the Spring transition to DST on driving fatigue of young male drivers [23], documenting a significant decrease in both driving-based and physiological measures of fatigue. This raises concern, as fatigue ranks among the most critical contributing factors to road accidents, accounting for 16–20 % of fatal crashes in the USA [[24], [25], [26]], and contributing to 15–20 % of serious accidents in Europe [27]. Driving fatigue can be subdivided into sleep-related fatigue, resulting from circadian desynchrony and sleep curtailment, and task-related fatigue, stemming from the act of driving itself and from environmental factors [28]. The latter can be further categorized into active task-related fatigue, associated with mental overload, and passive task-related fatigue, linked to conditions of mental underload, i.e. a reduction in attentional capacity that associates with prolonged reductions in mental workload [28].

The present study builds on our previous experience, providing further evidence of short-term sleep/circadian-related effects of DST on driving fatigue and studying its long-term effects, if any, for the first time. Participants were exposed to a combination of passive task-related fatigue (associated with a monotonous driving task at the simulator) and sleep-related fatigue (associated to DST). The chosen experimental design enabled us to separate and study these two distinct components.

## 2 Material and methods

### 2.1 Driving simulator experiment

#### 2.1.1 Experimental design and study overview

The study involved 40 participants in total, with 37 of them (10 females, age range 19–30, mean = 22.9) being able to complete all the required trials. Each trial involved driving on a monotonous highway scenario for 50 min. Eighteen participants (all males, age range 21–30, mean = 24.1) underwent the experiment in 2022, which consisted of two trials, the first taking place in the week preceding the Spring transition to DST, the second in the following; results from this experiment uncovered negative DST-related effects on driving fatigue [23]. Nineteen additional participants (10 females, age range 19–24, mean = 21.7) underwent the experiment in 2023, when an additional third trial was carried out in the fourth week after the Spring transition to DST. Thirteen of the 19 subjects of the 2023 cohort were also studied on two further occasions, i.e. in the week preceding and the one following the 2023 Autumn transition to civil time.

**Fig. 1:**
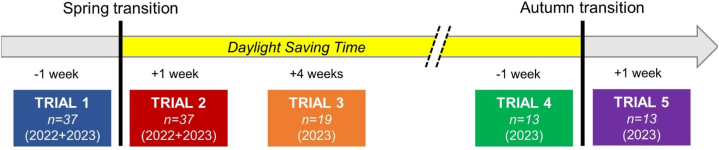
Visual representation of experimental design.

Data from the first two trials of the 2022 and 2023 experimental groups were cumulated to confirm the short-term effects of the DST transition, while controlling for possible confounding effects resulting from repeating the experiment in two different years on different individuals. Data from the three (or five, where available) trials of the 2023 experiment were used to investigate its long-term effects. The experimental design is visually represented in Fig. 1; all additional details are reported in Sections 2.1.2–2.1.6.

The 2022 experiment served as a pilot study, with its initial findings reported in Orsini et al. [23]; it exclusively involved young male drivers to maintain a homogeneous sample, a necessity stemming from the limited number of participants that could be recruited due to the strict time constraints of the experimental design. The additional group recruited in 2023 allowed us to confirm and generalize the findings of the pilot study, not only doubling the sample size but also including female drivers. Despite this expansion, the sample was kept homogeneous in terms of age, a decision that was influenced by the fact that driving fatigue is a particularly significant contributing factor to road accidents involving young drivers [[29], [30], [31]].

#### 2.1.2 Participants

A total of 40 participants were initially recruited, with 20 of them being enrolled in 2022 and 20 in 2023. Two participants from the 2022 experiment and one from the 2023 experiment were unable to complete all the required Trials and were therefore excluded from subsequent analyses. Thus, the final sample included 37 participants, whose sociodemographic and sleep characteristics are detailed in Table 1. Six further participants from the 2023 cohort did not return for Trials 4 and 5 in the Autumn, with a total of 13 participants performing all 5 Trials (Fig. 1).

**Table 1:** Sociodemographic and sleep (*vide infra*) features of participants; mean values (standard deviation in round brackets).

Variable	Experimental group (2022)	Experimental group (2023)	Experimental group (2022 + 2023)
N	18	19	37
Age [years]	24.1 (2.9)	21.7 (0.9)	22.9 (2.4)
Sex	100 % males	47 % males, 53 % females	73 % males, 27 % females
Driving experience [years]	5.5 (3.0)	3.1 (1.1)	4.3 (2.4)
Annual mileage [km]	10,053 (12,690)	5997 (3564)	8038 (9350)
Pittsburg Sleep Quality Index (0–21, abnormal >5)	4.3 (1.5)	4.4 (2.1)	4.3 (1.8)
Epworth Sleepiness Scale (0–24, abnormal ≥11)	5.8 (2.6)	5.2 (2.7)	5.5 (2.8)
Sleep duration (working days) [hh:mm]	07:33 (01:07)	07:45 (01:05)	07:41 (01:06)
Sleep duration (free days) [hh:mm]	08:16 (01:11)	08:19 (01:03)	08:18 (01:06)
Midsleep (working days) [hh:mm]	03:55 (00:59)	03:33 (00:56)	03:43 (00:58)
Midsleep (free days) [hh:mm]	05:22 (01:09)	04:29 (00:46)	04:53 (01:03)
Social jet lag [hh:mm]	01:27 (00:59)	00:55 (00:56)	01:10 (00:59)

Eye-tracking data from 2 participants in Trial 1, 3 in Trial 2, 2 in Trial 3, 2 in Trial 4, and 2 in Trial 5 was discarded due to eye-tracker calibration issues.

Inclusion criteria were:

• Normal or corrected-to normal vision (with contact lenses and not glasses due to incompatibility with the eye-tracker);
• ≥ 1 year since first obtainment of a full Italian driving license and ≥1000 km average annual mileage;
• No significant illnesses or diagnosed sleep disorders, and no sleep-inducing or psychoactive medication;
• No history of shiftwork;
• No previous experience with driving simulators.

Their formal sleep-wake evaluation at the time of the experiments confirmed no significant night sleep disturbance, no significant daytime sleepiness nor abnormalities in sleep duration (Table 1).

Participants were not asked to modify their habits in relation to sleep-wake patterns and alcohol/caffeine consumption. Participants were provided with a small monetary compensation upon completion of the experiment and were unaware of its specific objectives. The research protocol was granted ethical approval by the Ethics Committee for Psychological Research at the University of Padua (Protocol 4037, 24/03/2021), in adherence with the Code of Ethics of the World Medical Association [32]. Written informed consent was acquired from all participants.

#### 2.1.3 Subjective sleep-wake and fatigue assessment

Prior to the first trial day, participants were asked to complete the following questionnaires online:

The *Pittsburgh Sleep Quality Index (PSQI)* [33,34]. This encompasses 19 questions, which are used to generate seven components, each of which is scored from zero (best) to three (worst); these scores are then summated to provide the total PSQI score (range: 0–21); scores >5 indicate impaired sleep quality [33].

The *Epworth Sleepiness Scale (ESS)* [35,36]. Subjects are asked to evaluate their likelihood of ‘dozing off’ in eight different situations, on a scale of zero (unlikely) to three (very likely). These scores are then summated to provide the ESS score (range: 0–24); scores ≥11 indicate excessive daytime sleepiness [35].

The *ultra-short version of the Munich ChronoType Questionnaire (μMCTQ)* [37]. This encompasses 6-questions, which allow to calculate sleep duration, midsleep (i.e. the midpoint, expressed as clock time, between sleep onset and sleep offset on free and work/study days) and social jetlag (uncorrected difference between midsleep on free and work/study days).

On each trial day, participants were asked to complete the following questionnaires, before and after the driving task:

The *Stanford Sleepiness Scale (SSS)* [38]*.* This is a one-item self-reported questionnaire assessing subjective sleepiness on a 7-point scale. Scores >3 qualify participants as “sleepy” [39].

The *Samn-Perelli Fatigue Scale (SPF)* [40]. This is a one-item self-reported questionnaire assessing subjective fatigue [range 1 (“fully alert, wide awake”) to 7 (“completely exhausted, unable to function effectively”)]. Scores ≥5 are considered to be critical [41].

#### 2.1.4 Apparatus

**Fig. 2:**
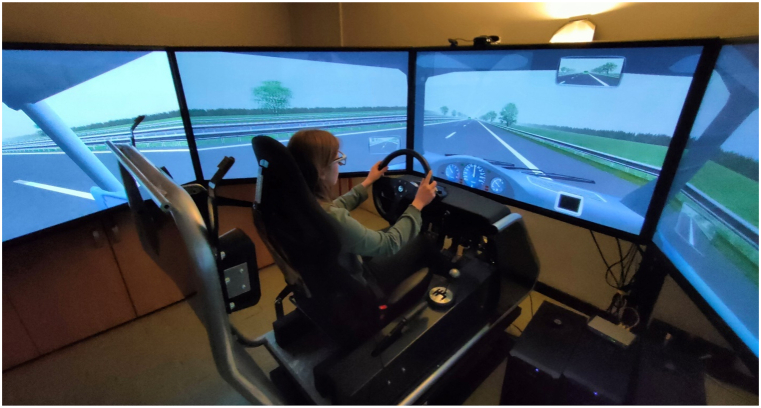
Driving simulator setup and virtual environment.

The experiments were carried out at the Mobility & Behavior Research Center (MoBe), using a dynamic driving simulator with 2 degrees of freedom, developed by StSoftware (Fig. 2). Previous validation [42,43] has established realism and reliability of the setup, which has been utilized in in several road safety studies [22,[44], [45], [46], [47], [48]]. The hardware configuration includes a seat, a force-feedback steering wheel, three pedals, a manual gearbox, and a handbrake. Five 60-inch full-HD monitors provide an immersive 330° by 45° field of view, complemented by six speakers. Operating at a sampling rate of 50 Hz, the simulation system captures 31 kinematic variables, including (position, speed, lateral position, acceleration, and steering wheel rotation, among others). In addition, a small virtual dashboard positioned in the lower-right corner of the screen (Fig. 2) serves as a means for conveying messages or signals to the driver. For the current experiment, the simulator was configured in a static mode to minimize activation by external stimuli.

During the driving task, eye gaze data were collected using the SMI eye-tracking glasses 2 Wireless (SensoMotoric Instruments, Germany), a non-invasive system designed to resemble ordinary glasses and equipped with HD cameras (resolution 1280 × 960p) to capture binocular gaze position and eye movement. Data acquisition occurred at a rate of 60 Hz, and extraction was carried out by the BeGaze software (SensoMotoric Instruments, Germany).

The driving simulator room was maintained at an average temperature between 20 °C and 22 °C, with an illuminance of 4 lx.

#### 2.1.5 Experimental procedure

The study consisted of five driving simulator trials within the same virtual environment on separate days. The first trial occurred during the week preceding the Spring transition to DST (March 21st – 25th, 2022; March 20th – 24th, 2023), the second in the following week (March 28th – April 1st, 2022; March 27th – 31st, 2023), the third in the fourth week after the transition (April 17th – 21st, 2023), the fourth in the week preceding the Autumn transition to civil time (October 23rd – 27th, 2023), and the fifth in the following week (October 30th – November 3rd, 2023). The experiments were all conducted in the afternoon, Monday to Friday, with four trials carried out each day, the first starting at 15:00 and the last one at 18:00. The selected time window was chosen to mitigate the potential influence of the “post-lunch dip” on driving fatigue [49]. These strict constraints limited the experimental group to a maximum of 20 participants per year. Each participant completed the three trials on the same weekday and at the same time.

Before the first trial, participants were instructed to complete online questionnaires providing sociodemographic, sleep quality and sleep timing information (please refer to Section 2.1.3). On Trial days, participants' perceived sleepiness and fatigue were assessed before and after the driving simulator test using the SSS and the SPF, respectively.

The driving task involved driving for 50 min in a monotonous highway environment. Participants were instructed to drive similarly to their usual driving behavior in a real-world setting. They were asked to verbally self-assess their level of sleepiness every 9 min using a 1–10 self-reported sleepiness scale (a modified version of the Karolinska Sleepiness Scale, KSS, with 1 being the lowest sleepiness level and 10 the highest), adapted from Åkerstedt et al. [50]. Participants were not provided with information regarding the trial duration beforehand. They were asked to switch off their phone and remove their wristwatch before commencing the experiment.

#### 2.1.6 Driving scenario

The driving scenario encompassed a straight two-lane two-carriageway segment spanning a length of 164 km. The carriageway featured a virtual banking effect that induced a slight inclination to veer towards the right; this required drivers to remain vigilant and apply compensatory steering adjustments to keep the vehicle within the designated lane. The simulation adopted daytime conditions with overcast skies, ensuring favorable visibility of up to 500 m. The prescribed speed limit was set at 130 km/h.

The simulated traffic conditions mirrored very light flow in both directions. In the primary direction, the traffic consisted of vehicles traveling at an equal or higher speed compared to that of the participant, thus disallowing active overtaking maneuvers. This deliberate design aimed to induce mental underload, to allow the investigation of passive task-related fatigue.

The scenario was identical in all experimental trials.

### 2.2 Variables analyzed

To investigate passive task-related driving fatigue, various metrics/indices were employed, encompassing subjective measures (SSS, SPF, KSS), along with an objective driving-based measure (the standard deviation of the lateral position, SDLP) and an objective physiological measure (PERcent of eye CLOSure, PERCLOS). In the current study, both SDLP and PERCLOS measurements were aggregated into 5-min intervals; i.e., the 50-min experiment was divided into ten discrete 5-min segments, and SDLP and PERCLOS values were computed within each segment.

The SDLP within a specified time interval was computed relative to the lane axis occupied by the participant's vehicle. SDLP has been demonstrated to be a valid tool to objectively assess driving fatigue [[50], [51], [52]]. As drivers experience increasing levels of fatigue, their ability to accurately perceive their position within the lane diminishes. As a consequence, their corrective responses to maintain the vehicle's position and trajectory become less frequent and more pronounced, leading to greater lateral swaying with reference to the lane central axis [53]. Therefore, higher levels of driving fatigue are associated with larger SDLP values, which indicate a reduced ability to maintain a consistent position within the lane.

PERCLOS is a measure used to assess drowsiness by quantifying the amount of time the eyelids are closed above a predefined threshold within a specific time period. It is widely used as an indicator of drowsiness [[54], [55], [56]]. In accordance with the existing literature [57,58], this study used a conservative 80 % threshold to define eyelid closure. Thus, PERCLOS was calculated as the percentage of time during which the pupil was covered by more than 80 %, relative to each participant's detected maximum opening, within each time interval. The resulting ratio provides insights into the drivers' alertness, with higher PERCLOS values indicating higher levels of driving fatigue.

### 2.3 Statistical analyses

Separate analyses were conducted for each of the five dependent variables considered: SDLP, PERCLOS, SSS, SPF, and KSS.

Linear mixed-effect models (LMMs) were used for the analysis of scalar dependent variables (SDLP and PERCLOS) [59]. The fixed effect factors included Trial (2 levels for the short-term analysis, 3 or 5 levels for the long-term analysis) and Time (with 10 levels corresponding to the 10 5-min segments into which the experiment was discretized). Year was an additional fixed factor in the analysis of the short-term effects. Time was considered as a marker of passive task-related fatigue, Trial to assess the DST-related effects, and Year to account for any potential confounding effects resulting from repeating the experiment in two different years on different individuals. The ID of each participant was treated as a random factor.

LMMs were chosen due to their ability to handle repeated-measures designs with missing data, thus maximizing the available information despite dropouts. Nevertheless, for additional robustness, the analyses on long-term effects were replicated by exclusively considering data from participants who completed all five trials.

The Satterthwaite approximation was used to compute degrees of freedom for reported F-statistics, allowing for unequal variances [60,61]. Due to concerns regarding the control of Type I errors and the complexity associated with interpreting factors involving multiple levels and interactions [59], we chose not to perform statistical inference directly on the parameter estimates. Thus, these are not reported.

QQplot diagnostics was used to test normality of LMMs residuals. LMMs are known to be robust to violations of distributional assumptions [62]. Nevertheless, to ensure additional robustness, when such assumption was not met, the dependent variable was transformed prior to model fitting using a Box-Cox transformation [63]. In Section 3, all reported marginal means were back-transformed for ease of interpretation.

Cumulative link mixed-effect models (CLMMs) were used for the analysis of ordinal dependent variables (SSS, SPF, KSS) [64,65]. These models had a structure similar to the previously described LMMs, incorporating Trial and Time as fixed factors and participant ID as a random factor. For the Time factor, there were 2 levels for SSS and SPF (“before” and “after”) and 5 levels for KSS (corresponding to the five self-evaluations conducted during the driving task).

In the *post hoc* tests, *p*-value adjustments were carried out with the Tukey method. All statistical analyses were performed using the R software. The following R packages were used:“lme4” [66] for estimating the LMMs, “ordinal” [67] for estimating the CLMMs,“lmerTest” [61] and “emmeans” [68] for the *post hoc* analyses. Significance level was set at *α* = 0.05, p-values between .05 and .075 were reported as marginally significant.

## 3 Results

To investigate driving fatigue, several metrics/indices were employed, encompassing one objective driving-based measure (SDLP, registered by the driving simulator), one objective physiological measure (PERCLOS, recorded with an eye-tracker) and questionnaire-based subjective measures.

### 3.1 Short-term effects of DST on driving-fatigue

The short-term effects of the Spring transition to DST on objective measurements of driving fatigue were analyzed by linear mixed-effects models (LMMs). This analysis considered not only the data collected during the 2023 experiment but also those from the pilot study conducted in 2022 in identical conditions, as reported in Orsini et al. [23]. This combined dataset included a total of 37 participants.

Two separate models were built, one for investigating SDLP, the other for PERCLOS. Fixed-effects factors considered in the models were Time (categorized into 10 levels of 5-min intervals within the 50-min task), Trial (with 2 levels: Trial 1 occurring in the week preceding the DST transition and Trial 2 in the week after), Year (with 2 levels: 2022 and 2023 experiments), and their interactions.

**Fig. 3:**
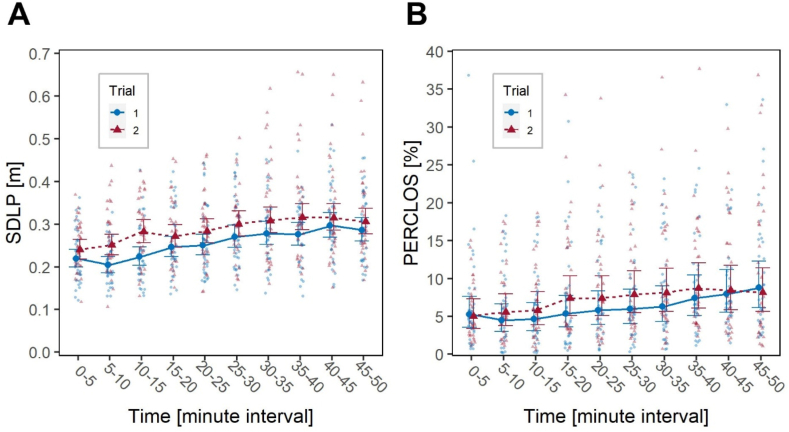
Short-term impact of the transition to DST. Effect of factors Trial and Time on: (**A**) SDLP and (**B**) PERCLOS. Large blue circles (Trial 1), and large red triangles (Trial 2) represent marginal means, bars are 95 % confidence intervals of marginal means; background, smaller and lighter blue circles (Trial 1), and red triangles (Trial 2) are individual observations. (For interpretation of the references to colour in this figure legend, the reader is referred to the Web version of this article.)

The factor Time had a significant effect on both SDLP, *F*(9, 673.01) = 24.9, *p* < .001, and PERCLOS, *F*(9, 612.70) = 8.7, *p* < .001, indicating an overall, progressive increase in driving fatigue throughout the duration of the driving task (Fig. 3).

The factor Trial also had a significant effect on both SDLP, *F*(1, 673.68) = 75.7, *p* < .001, and PERCLOS, *F*(1, 621.59) = 12.3, *p* < .001. Specifically, participants exhibited higher SDLP values in the post-DST compared to the pre-DST trial (estimated marginal means, EMM: 0.286 m vs. 0.253 m) and higher PERCLOS (EMM: 6.74 % vs. 5.75 %), indicating an increase in fatigue in the week following the DST transition. There was no statistical significance of the interaction Time*Trial, suggesting that SDLP and PERCLOS trends evolved similarly throughout the driving task.

Neither the factor Year nor any of its interactions with other factors were significant, indicating that the observed results were not influenced by any confounding factors related to the two experimental groups.

To analyze the effects of the DST transition on participants’ subjective perception, SSS, SPF, KSS were used. Due to the ordinal nature of the dependent variables, effects on subjective measurements of fatigue were investigated by CLMMs. As for previous analyses, each dependent variable was treated separately. Time, Trial and Year were considered as fixed effect factors, and subject ID as a random grouping factor. When investigating SSS and SPF, Trial had two levels, “before” and “after”, as the questionnaires were administered twice. In the case of the KSS, Trial had 5 levels, i.e. each of the five times participants were asked to report their level of sleepiness (minutes 9, 18, 27, 36, 45).

**Fig. 4:**
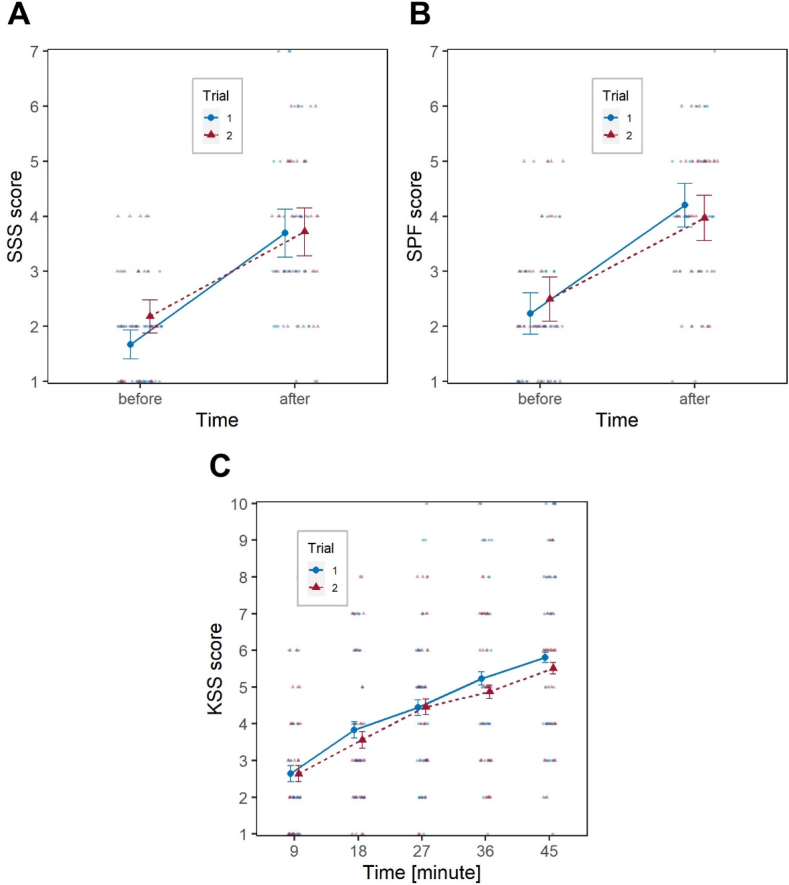
Short-term impact of the transition to DST. Effect of the factors Trial and Time on: (**A**) SSS; (**B**) SPF; (**C**) KSS. Large blue circles (Trial 1), and large red triangles (Trial 2) represent marginal means, bars are 95 % confidence intervals of marginal means; background smaller and lighter blue circles (Trial 1) and red triangles (Trial 2) are individual observations. (For interpretation of the references to colour in this figure legend, the reader is referred to the Web version of this article.)

As expected, the factor Time had significant effects on all variables (*χ*^2^_1_ = 82.6, *p* < .001 on SSS; *χ*^2^_1_ = 65.8, *p* < .001 of SPF, *χ*^2^_4_ = 170.6, *p* < .001 on KSS), with participants demonstrating an awareness of their decreasing alertness over the course of the driving task (Fig. 4).

The factor Trial had a significant effect on SSS, *χ*^2^_1_ = 4.0, *p* = .046, and its interaction with Time was also significant, *χ*^2^_1_ = 4.3, *p* = .038. In further detail, participants tended to report a higher level of sleepiness before starting the task in the second trial, as documented by *post hoc* tests, *z* = 2.91, *p* = .019. However, they subsequently reported a smaller decrease in alertness throughout the task, which contrasts with the observations made using objective measurements. The factor Trial and the interaction Time*Trial did not have significant effects on SPF and KSS. Participants did not report any significant difference in fatigue levels before and after the task, or in sleepiness levels throughout the task. The factor Year and its interactions with Time and Trial were not significant on any variable.

### 3.2 Long-term effects of DST on driving-fatigue

To investigate the long-term effects of the Spring transition to DST, data from the 19 participants who carried out the experiment in 2023 were analyzed, with the same statistical approach. Fixed effect factors were Time and Trial. In this case, the factor Trial had three levels, including Trial 3, which took place in the fourth week after the transition (Fig. 1).

**Fig. 5:**
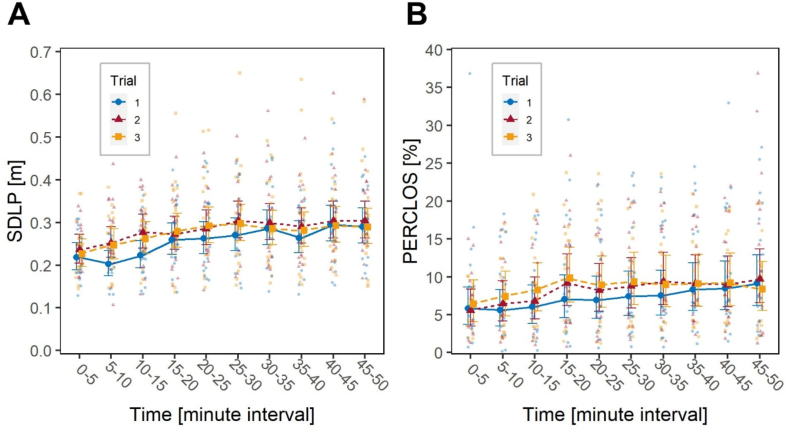
Long-term impact of the transition to DST. Effect of factors Trial and Time on: (**A**) SDLP and (**B**) PERCLOS. Large blue circles (Trial 1), large red triangles (Trial 2) and large orange squares (Trial 3) represent marginal means, bars are 95 % confidence intervals of marginal means; background smaller and lighter blue circles (Trial 1), red triangles (Trial 2) and orange squares (Trial 3) are individual observations. (For interpretation of the references to colour in this figure legend, the reader is referred to the Web version of this article.)

A significant effect of the factor Time on SDLP was observed, *F*(9, 522) = 13.6, *p* < .001, indicating that the level of fatigue, as expected, tended to increase throughout the driving task. The factor Trial was also significant, *F*(2, 522) = 12.4, *p* < .001, indicating that the observed level of fatigue was different in the three trials. *Post hoc* analysis showed a significant difference between the SDLP recorded during Trial 1 and Trial 2, *t*(522) = 4.80, *p* < .001, and between Trial 1 and Trial 3, *t*(522) = 3.51, *p* = .001, but not between Trials 2 and 3. This is presented in Fig. 5A, where a distinct increase in SDLP is observed between the first and the second trial, while in Trial 3 SDLP tends to overlap with that of Trial 2. No statistical significance of the interaction Time*Trial was observed, meaning that the three SDLP trends evolved similarly throughout the driving task.

These findings were paralleled by those obtained from the same analysis performed on PERCLOS. Time and Trial showed significant effects, respectively *F*(9, 457.86) = 5.8, *p* < .001 and *F*(2, 459.69) = 6.8, *p* = .001, while their interaction did not. *Post hoc* analyses confirmed statistical differences between Trials 1 and 2, *t*(460) = 2.60, *p* = .026, and between Trials 1 and 3, *t*(460) = 3.54, *p* = .001, but not between Trials 2 and 3. Visually, Fig. 5B presents PERCLOS trends that are consistent with the SDLP trends presented in Fig. 5A.

Regarding the subjective measurement of fatigue, as expected, the factor Time had a significant effect on both SSS, *χ*^2^_1_ = 48.7, *p* < .001, and SPF, *χ*^2^_1_ = 55.7, *p* < .001, with participants reporting higher values of sleepiness and fatigue after the driving task. The interaction Time*Trial was also significant for both indicators, *χ*^2^_2_ = 6.1, *p* = .047 and *χ*^2^_2_ = 8.6, *p* = .014, respectively, while Trial itself was not significant.

**Fig. 6:**
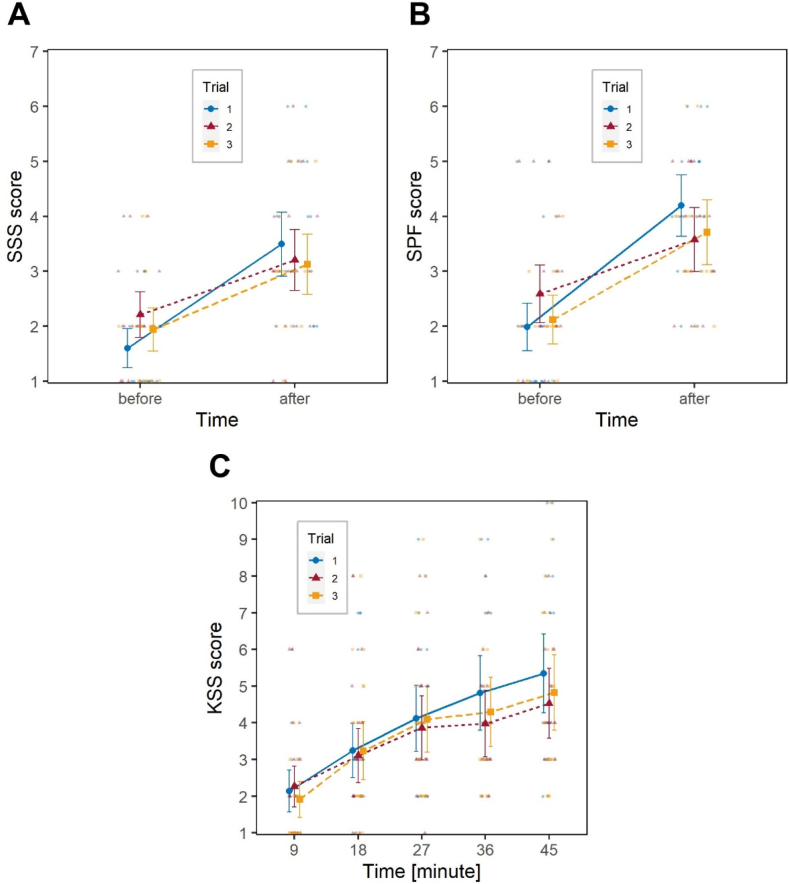
Long-term impact of the transition to DST. Effect of the factors Trial and Time on: (**A**) SSS; (**B**) SPF; (**C**) KSS. Large blue circles (Trial 1), large red triangles (Trial 2) and large orange squares (Trial 3) represent marginal means, bars are 95 % confidence intervals of marginal means; background smaller and lighter blue circles (Trial 1), red triangles (Trial 2) and orange squares (Trial 3) are individual observations. (For interpretation of the references to colour in this figure legend, the reader is referred to the Web version of this article.)

Fig. 6A and B show that the increase in perceived sleepiness/fatigue after the driving task was higher in Trial 1 compared to the two trails performed after the transition to DST. The *post hoc* analysis documented no significant differences in SSS and SPF scores among the three Trials, neither before nor after the driving task, apart from a marginally significant difference between the SSS reported before the driving task between Trial 1 and Trial 2, *z* = 2.19, *p* = .073, the SSS score being higher in Trial 2, which is consistent with what observed short-term.

As for the dependent variable KSS, again Time had a significant effect, *χ*^2^_4_ = 127.8, *p* < .001, but neither Trial nor the interaction Time*Trial were significant. Fig. 6C illustrates how in each of the three Trials participants reported progressively increasing sleepiness, with no substantial variations across Trials.

The analyses were then replicated, incorporating data from the 13 participants who also returned for Trials 4 and 5, in the weeks preceding and following the Autumn transition to civil time, confirming significant effects of the factors Time, *F*(9, 762.06) = 16.9, *p* < .001, and Trial, *F*(4, 763.09) = 14.9, *p* < .001, on SDLP.

**Fig. 7:**
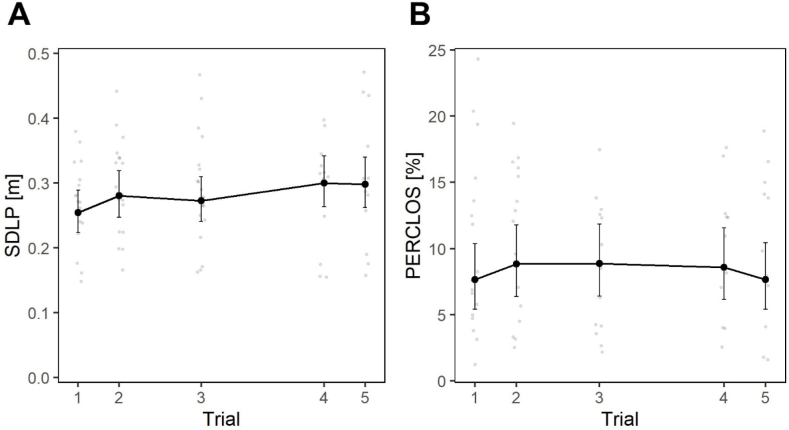
Long-term effect of the transition to DST. Effect of the factors Trial on: (**A**) SDLP; (**B**) PERCLOS. Large circles represent marginal means, bars are 95 % confidence intervals of marginal means, background smaller and lighter circles are individual observations.

*Post hoc* analyses confirmed statistical differences between Trial 1 and Trial 2, *t*(762) = 4.47, *p* < .001, and between Trial 1 and Trial 3, *t*(762) = 3.32, *p* = .011, and the absence of any difference between Trials 2 and 3. In addition, they showed that Trial 4, *t*(764) = 6.59, *p* < .001, and Trial 5, *t*(764) = 6.35, *p* < .001, were both statistically different from Trial 1 (EMM = 0.254 m), exhibiting an upward trend (expressing worse driving performance) with EMMs of 0.300 m and 0.298 m, respectively. In absolute terms, these values were even higher than those recorded in Trial 2 and Trial 3 (EMM = 0.280 m for Trial 2; EMM = 0.273 for Trial 3), as can be observed in Fig. 7A. A marginally statistical difference between Trials 2 and 4, *t*(764) = 2.69, *p* = .057, and statistical differences between Trials 3 and 4, *t*(764) = 3.77, *p* = .002, and between Trials 3 and 5, *t*(764) = 3.52, *p* = .004 were observed. For additional robustness, the same analysis was repeated including only the 13 participants who completed all 5 Trials, and this confirmed the main effects and the statistically significant increase in SDLP in Trials 4 and 5 compared to the preceding Trials.

The analysis on PERCLOS confirmed the results obtained in the previous section, with a significant effect of Time, *F*(9, 638.95) = 7.3, *p* < .001, and Trial, *F*(4, 640.86) = 3.8, *p* = .004. However, these were not entirely consistent with those observed on SDLP. Indeed, the *post hoc* analysis revealed statistical differences only between Trials 1 and 2, *t*(641) = 2.87, *p* = .035, and between Trials 1 and 3, *t*(641) = 2.94, *p* = .028, while Trials 4 and 5 were not statistically different from any other Trial. In terms of EMMs, as can be observed in Fig. 7B, Trial 4 showed a PERCLOS which was higher than that of Trial 1 (+0.96 %), but lower than that of Trials 2 (−0.24 %) and 3 (−0.27 %). In Trial 5, PERCLOS returned to values which were very similar to those of Trial 1 (+0.03 %).

In terms of subjective measurements of fatigue, the factor Time was statistically significant for SSS, *χ*^2^_1_ = 66.8, *p* < .001, SPF, *χ*^2^_1_ = 83.7, *p* < .001, and KSS, *χ*^2^_4_ = 104.5, *p* < .001, while the factor Trial was not significant for either of them, indicating that fatigue perception did not substantially change throughout the Trials, as also partly observed in the previous sections. These findings were confirmed when the analysis was restricted to the 13 participants who completed all five Trials.

## 4 Discussion

Driving fatigue was analyzed with a range of indicators which included objective measurements (SDLP and PERCLOS) and subjective indices (SSS, SPF, KSS scores). The observation that the factor Time had a significant effect on SDLP and PERCLOS in all analyses suggests that participants’ fatigue levels increased throughout the driving task. This outcome was expected, as the driving scenario was intentionally designed to produce mental underload. In this sense, the factor Time can be considered a marker of passive task-related fatigue, as defined by May & Baldwin [28]. Notably, in all Trials in this study, SDLP values largely exceeded the 0.25 m threshold defined by Brookhuis et al. [69] as a criterion to detect driving impairment, thus confirming that the experimental design produced the intended results.

The significant effect of Trial both short- and long-term indicates that the values of SDLP and PERCLOS differed across Trials, and more specifically they worsened. As Trials took place either before or after the Spring transition to DST, this factor can be regarded as a marker of DST-related driving fatigue. It should be noted that even if any familiarization/learning effect occurred (which we have previously excluded by comparing the 2022 cohort with a historical cohort of pertinent, matched controls who also underwent the two trials on exactly the same driving scenario without any time change in between [23,70]), this would have reasonably mitigated or masked the negative effect of DST. One might argue that the differences observed in the Trials could be attributed to other confounding factors, possibly including changes in photoperiod and other time-dependent environmental factors, which would not affect the experiment itself but would, of course, affect the participant's daily life around the time of the experiment. These, if any, would be more likely to impinge on Trials 4 and 5, which are further in time and in environmental conditions compared to the first three Trials.

Albeit being to some extent redundant, the results of this study confirm the negative short-term effects observed in our pilot study [23]. It is important to highlight that, by contrast to that earlier study, the 2023 cohort included both male and female participants, suggesting that the adverse short-term effects of the Spring transition to DST impact drivers regardless of sex. Given that the analysis of short-term effects revealed no significant impact of the factor Year on any of the dependent variables, one can be confident that no confounding effects or other factors related to participants’ features in the two groups influenced the overall conclusions regarding the effect of DST on driving fatigue. It is worth noting that all participants involved were healthy and had no sleep issues (please refer to Table 1). Since these drivers may represent a significant proportion of the general population, this observation further supports the hypothesis that DST can lead to serious road safety issues.

In absolute terms, SDLP increased by 3.3 cm (corresponding to a 13 % relative increase) and PERCLOS by 0.99 % in Trial 2. The magnitude of such effect is comparable, for example, to that associated with driving 0.5–2 h after the administration of 0.5 g/L alcohol [71,72] or 8.5–10 h after the administration of 7.5 mg of the sleep-inducing medication zopiclone [73,74] (∼2.6 cm SDLP, according to the meta-analysis by Vinckenbosch et al. [52], corresponding to a 13 % relative increase), and also of that associated with driving after a night of partial night sleep deprivation (2.5 cm SDLP, corresponding to a 11 % relative increase, and 0.43 % PERCLOS [75]). Several driving simulator-based studies have investigated the effects of alcohol on SDLP [76], although only a minority of them used driving simulators validated with alcohol [[77], [78], [79]], showing SDLP increases ranging from +2 cm (12 % relative increase) [78] to +6.6 cm (22 % relative increase) [77]. It should be noted that, although the simulator we used has been validated in terms of speeding [42] and gap-acceptance behaviour [43], it has never been specifically validated for any type of impaired driving. Therefore, the comparisons reported here should be interpreted with caution.

Analyses also revealed a significant long-term effect, which was comparable in magnitude to that observed short-term. In Trial 3, participants exhibited significantly higher values of both SDLP and PERCLOS compared to Trial 1, with no significant change from Trial 2. This is an important observation, as it suggests that the effects of DST on driving fatigue extend beyond the week immediately after the transition, persisting for at least one month. Further, the exploratory analysis conducted immediately prior to and after the Autum transition to DST revealed that not only was driving fatigue significantly worse in these trials compared to Trial 1, but it was also slightly worse than that ofd Trials 2 and 3. Besides supporting the contention that DST-related circadian misalignment has effects on performance that last as long as DST is in place, it also suggests that such effects are likely to be cumulative [3]. The analysis on PERCLOS exhibited a different time dynamic, with a small improvement in Trial 4, and a significant one in Trial 5, with a return to the levels of Trial 1. This might be explained by the fact that the effects of driving fatigue measured by SDLP are probably an expression of its effects on complex cognitive functions and their combination, and they may therefore require a significant amount of time to revert once they have accumulated, explaining the similarity between Trials 4 and 5, immediately before and immediately after the Autumn return to civil time. By contrast, eyelid closure may change more rapidly in relation to decreased/increased sleep duration, and may vary less than SDLP (in terms of magnitude) in response to small changes in fatigue levels [80]. This hypothesis is supported by the time-course of PERCLOS levels (Fig. 7B), which rapidly deteriorated after Trial 1, and then rapidly returned to baseline levels in Trial 5, suggesting a quicker response to daily misalignment or lack thereof, rather than a cumulative effect as that observed for SDLP. Indeed, there is no reason to expect that a physiological index such as PERCLOS would have the same time-course as a driving-simulator based index, which is the outcome of a complex network of cognitive functions, possibly even including eyelid movements.

The fact that no significant interaction between the factors Time and Trial was observed is also remarkable, because it indicates no combination in the effects of passive task-related and DST-related fatigue, i.e., that the negative effects of the transition to DST do not necessarily emerge in mental underload situations. Fig. 3, Fig. 5 show that, if anything, the effect tends to be more pronounced at the beginning rather than at the end of the driving task. This aligns with our previous work [22], documenting worsened overall driving performance in a relatively short (∼15 min) experimental route in the week following the Spring transition to DST.

Analyses performed on subjective indicators of fatigue confirmed that participants felt more fatigued after the driving task, but also documented a lack of awareness of the objectively-measured decrease in performance after DST. By contrast, the short-term analysis carried out on SSS and the long-term analysis on SSS and SPF showed a significant effect of the interaction Time*Trial, indicating, as can be observed in Fig. 4, Fig. 6A–B, that participants felt proportionally less sleepy and less fatigued after the driving task in Trials 2 and 3 compared to Trial 1. This may either be related to Trial 1 being generally more taxing than subsequent Trials, simply because of total lack of familiarity with the task. An alternative explanation may be that as circadian desynchrony accumulates over the DST period, the participants’ insight into their actual performance decreases, which is known to occur when sleep is curtailed [81,82]. This effect is concerning in terms of its road safety implications, as it may prevent drivers from engaging in compensatory or fatigue-coping strategies.

## 5 Conclusion

The key findings of this study can be summarized as follows.

1. The Spring transition to DST had a significant negative impact on objectively-measured driving fatigue in the short-term, confirming our previous findings [23].
2. DST *per se* also had a significant, negative long-term effect, as the increase in objectively-measured driving fatigue persisted in the fourth week after the transition, with the same magnitude. Further worsening of these parameters was observed at the very end of the DST period, and also in the week after the return to civil time in Autumn.
3. Participants were unaware of this increase in driving fatigue, as indicated by the SSS and SPF scores reported before and after each trial, as well as the KSS scores reported during the driving tasks.

Albeit preliminary, our findings regarding the long-terms effects of DST – which are perfectly reasonable in terms of chronophysiology and chronopathology but had never been directly tested before – are of great relevance, and could suggest that DST is a contributing factor to the higher number of road fatalities [83] and sleep-related fatal crashes [84], which have been observed during the Summer months in several nations adopting DST. Further, as drivers are unaware of the decline in their performance, informative campaigns on the effects of DST on driving - and how to manage them - targeting the general population may represent a valuable mitigation strategy.

Future studies should probably investigate the DST effects on other age groups, also keeping into account of chronotype and of the course of sleep quality/timing indices over the DST months. In addition, extension of the study on the effects of DST on driving performance to naturalistic settings may be worthwhile. Lastly, we designed this study to be ecological, and therefore we did not control for sleep-wake patterns and alcohol/caffeine consumption during or on the day of study. Future research should investigate how these may modulate the effects of DST on driving performance.

## Ethics statement

The research protocol was granted ethical approval by the Ethics Committee for Psychological Research at the 10.13039/501100003500University of Padua (Protocol 4037, 24/03/2021). Written informed consent was acquired from all participants.

## Data availability statement

Data associated with the study has not been deposited into a publicly available repository. Data will be made available on request.

## CRediT authorship contribution statement

**Federico Orsini:** Writing – original draft, Visualization, Software, Methodology, Investigation, Formal analysis, Data curation, Conceptualization. **Esther Dingena Domenie:** Writing – review & editing, Visualization, Methodology, Investigation, Data curation. **Lisa Zarantonello:** Writing – review & editing, Methodology, Data curation. **Rodolfo Costa:** Writing – original draft, Supervision, Methodology, Funding acquisition, Conceptualization. **Sara Montagnese:** Writing – original draft, Supervision, Resources, Methodology, Funding acquisition, Conceptualization. **Riccardo Rossi:** Writing – review & editing, Validation, Supervision, Resources, Methodology, Funding acquisition, Conceptualization.

## Declaration of competing interest

The authors declare no competing interests.

## References

[1] Roenneberg T., Winnebeck E.C., Klerman E.B. (2019). Daylight saving time and artificial time zones - a battle between biological and social times. Front. Physiol..

[2] Ferrazzi E., Romualdi C., Ocello M., Frighetto G., Turco M., Vigolo S., Fabris F., Angeli P., Vettore G., Costa R., Montagnese S. (2018). Changes in accident & emergency visits and return visits in relation to the enforcement of daylight saving time and photoperiod. J. Biol. Rhythm..

[3] Johnson K.G., Malow B.A. (2022). Daylight saving time: neurological and neuropsychological implications. Curr. Sleep Med. Reports.

[4] Antle M.C. (2023). The controversy over daylight saving time: evidence for and against. Curr. Opin. Pulm. Med..

[5] Coate D., Markowitz S. (2004). The effects of daylight and daylight saving time on US pedestrian fatalities and motor vehicle occupant fatalities. Accid. Anal. Prev..

[6] Ellis W.A., FitzGibbon S.I., Barth B.J., Niehaus A.C., David G.K., Taylor B.D., Matsushige H., Melzer A., Bercovitch F.B., Carrick F., Jones D.N., Dexter C., Gillett A., Predavec M., Lunney D., Wilson R.S. (2016). Daylight saving time can decrease the frequency of wildlife-vehicle collisions. Biol. Lett..

[7] Lahti T.A., Leppämäki S., Lönnqvist J., Partonen T. (2006). Transition to daylight saving time reduces sleep duration plus sleep efficiency of the deprived sleep. Neurosci. Lett..

[8] Lahti T., Nysten E., Haukka J., Sulander P., Partonen T. (2010). Daylight saving time transitions and road traffic accidents. J. Environ. Public Health.

[9] Sullivan J.M., Flannagan M.J. (2002). The role of ambient light level in fatal crashes: inferences from daylight saving time transitions. Accid. Anal. Prev..

[10] Carey R.N., Sarma K.M. (2017). Impact of daylight saving time on road traffic collision risk: a systematic review. BMJ Open.

[11] Abeyrathna W.A.N.U., Langen T.A. (2021). Effect of Daylight Saving Time clock shifts on white-tailed deer-vehicle collision rates. J. Environ. Manag..

[12] Bünnings C., Schiele V. (2021). Spring forward, don't fall back: the effect of daylight saving time on road safety. Rev. Econ. Stat..

[13] Singh R., Sood R., Graham D.J. (2022). Road traffic casualties in Great Britain at daylight savings time transitions: a causal regression discontinuity design analysis. BMJ Open.

[14] Zhou R., Li Y. (2022). Traffic crash changes following transitions between daylight saving time and standard time in the United States: new evidence for public policy making. J. Saf. Res..

[15] Cunningham C.X., Nuñez T.A., Hentati Y., Sullender B., Breen C., Ganz T.R., Kreling S.E.S., Shively K.A., Reese E., Miles J., Prugh L.R. (2022). Permanent daylight saving time would reduce deer-vehicle collisions. Curr. Biol..

[16] Molina J.E., Kitali A., Alluri P. (2023). Relationship between daylight saving time and traffic crashes in Florida. Transp. Res. Rec. J. Transp. Res. Board.

[17] Fritz J., VoPham T., Wright K.P., Vetter C. (2020). A chronobiological evaluation of the acute effects of daylight saving time on traffic accident risk. Curr. Biol..

[18] Prats-Uribe A., Tobías A., Prieto-Alhambra D. (2018). Excess risk of fatal road traffic accidents on the day of daylight saving time change. Epidemiology.

[19] Robb D., Barnes T. (2018). Accident rates and the impact of daylight saving time transitions. Accid. Anal. Prev..

[20] James J. (2022). Let there be light: daylight saving time and road traffic collisions. Econ. Inq..

[21] Teke C., Kurtoğlu Çelik G., Yıldırım Ç., Şener A., Tanrıverdi F., Kahraman F.A., Gökhan Ş. (2021). Assessment of the number of admissions for road traffic collisions and severity of injury in daylight saving time and permanent daylight saving time periods. Int. J. Clin. Pract..

[22] Orsini F., Zarantonello L., Costa R., Rossi R., Montagnese S. (2022). Driving simulator performance worsens after the spring transition to daylight saving time. iScience.

[23] Orsini F., Giusti G., Zarantonello L., Costa R., Montagnese S., Rossi R. (2023). Driving fatigue increases after the Spring transition to Daylight Saving Time in young male drivers: a pilot study. Transport. Res. F Traffic Psychol. Behav..

[24] Owens J., Dingus T.A., Guo F., Fang Y., Perez M., McClafferty J., Tefft B. (2018). Prevalence of drowsy-driving crashes: estimates from a large-scale naturalistic driving study, AAA found. Traffic Saf..

[25] Tefft B.C. (2014). Prevalence of motor vehicle crashes involving drowsy drivers, United States, 2009-2013, AAA found. Traffic safety. https://aaafoundation.org/prevalence-motor-vehicle-crashes-involving-drowsy-drivers-united-states-2009-2013/.

[26] Tefft B.C. (2012). Prevalence of motor vehicle crashes involving drowsy drivers, United States, 1999-2008. Accid. Anal. Prev..

[27] European Road Safety Observatory (2021). Road safety thematic report-fatigue. European road safety observatory. https://www.swov.nl/en/facts-figures/factsheet/fatigue.

[28] May J.F., Baldwin C.L. (2009). Driver fatigue: the importance of identifying causal factors of fatigue when considering detection and countermeasure technologies. Transport. Res. F Traffic Psychol. Behav..

[29] Li R., Su W., Lu Z. (2017). Physiological signal analysis for fatigue level of experienced and inexperienced drivers. Traffic Inj. Prev..

[30] Martiniuk A.L.C., Senserrick T., Lo S., Williamson A., Du W., Grunstein R.R., Woodward M., Glozier N., Stevenson M., Norton R., Ivers R.Q. (2013). Sleep-deprived young drivers and the risk for crash the drive prospective cohort study. JAMA Pediatr..

[31] Zhang G., Yau K.K.W., Zhang X., Li Y. (2016). Traffic accidents involving fatigue driving and their extent of casualties. Accid. Anal. Prev..

[32] World Medical Association (2013). World Medical Association declaration of Helsinki: Ethical principles for medical research involving human subjects. JAMA, J. Am. Med. Assoc..

[33] Buysse D.J., Reynolds C.F., Monk T.H., Berman S.R., Kupfer D.J. (1989). The Pittsburgh sleep quality index: a new instrument for psychiatric practice and research. Psychiatr. Res..

[34] Curcio G., Tempesta D., Scarlata S., Marzano C., Moroni F., Rossini P.M., Ferrara M., De Gennaro L. (2013). Validity of the Italian version of the Pittsburgh sleep quality index (PSQI). Neurol. Sci..

[35] Johns M.W. (1991). A new method for measuring daytime sleepiness: the Epworth sleepiness scale. Sleep.

[36] Vignatelli L., Plazzi G., Barbato A., Ferini-Strambi L., Manni R., Pompei F., D'Alessandro R., Brancasi B., Misceo S., Puca F., Savarese M., Servalli C., Ubiali E., Viscardi M., Vetrugno R., Buzzi G., Cirignotta F., Mostacci B., Sancisi E., Fassari V., Scrofani A., Beelke M., Ferrillo F., Nobili L., Costa C., Di Perri R., Raffaele M., Landi C., Rossi M., Spaggiari C., Terzano M.G., Manni R., Sartori I., Zanotta N., Bonnani E., Indice A., Murri L., Guazzelli M., Palagini L., Panicucci P., Antonini G., Bruni O., Ceschini V., Gragnani F., Miano S., Della Marca G., Farina B., Mennuni G.F., Cosentino F., Ferri R., Bergonzi P., Marinig R., Pauletto G., Dolso P.L., Gigli G.L. (2003). Italian version of the Epworth sleepiness scale: external validity. Neurol. Sci..

[37] Ghotbi N., Pilz L.K., Winnebeck E.C., Vetter C., Zerbini G., Lenssen D., Frighetto G., Salamanca M., Costa R., Montagnese S., Roenneberg T. (2020). The μMCTQ: an ultra-short version of the Munich ChronoType questionnaire. J. Biol. Rhythm..

[38] Hoddes E., Zarcone V., Smythe H., Phillips R., Dement W.C. (1973). Quantification of sleepiness: a new approach. Psychophysiology.

[39] Berry R.B., Wagner M.H. (2014).

[40] Samn S., Perelli L. (1982). Estimating aircrew fatigue: a technique with application to airlift operations, usaf sch. Méd..

[41] Caa S.R.G. (2005). Aircrew fatigue: a review of research undertaken on behalf of the UK civil. Aviation Authority.

[42] Rossi R., Gastaldi M., Gecchele G., Biondi F., Mulatti C. (2014). Traffic-calming measures affecting perceived speed in approaching bends: on-field validated virtual environment. Transport. Res. Rec..

[43] Rossi R., Meneguzzer C., Orsini F., Gastaldi M. (2020). Gap-acceptance behavior at roundabouts: validation of a driving simulator environment using field observations. Transport. Res. Procedia.

[44] Orsini F., Gecchele G., Gastaldi M., Rossi R. (2019). Collision prediction in roundabouts: a comparative study of extreme value theory approaches. Transp. A Transp. Sci..

[45] Rossi R., Orsini F., Tagliabue M., Di Stasi L.L., De Cet G., Gastaldi M. (2021). Evaluating the impact of real-time coaching programs on drivers overtaking cyclists. Transport. Res. F Traffic Psychol. Behav..

[46] Orsini F., Baldassa A., Grassi M., Cellini N., Rossi R. (2024). Music as a countermeasure to fatigue: a driving simulator study. Transport. Res. F Traffic Psychol. Behav..

[47] Rossi R., Gastaldi M., Biondi F., Mulatti C. (2012). Evaluating the impact of processing spoken words on driving. Transport. Res. Rec..

[48] Biondi F.N., Rossi R., Gastaldi M., Orsini F., Mulatti C. (2020). Precision teaching to improve drivers' lane maintenance. J. Saf. Res..

[49] Zhang H., Yan X., Wu C., Qiu T. (2014). Effect of circadian rhythms and driving duration on fatigue level and driving performance of professional drivers. Transport. Res. Rec..

[50] Åkerstedt T., Ingre M., Kecklund G., Anund A., Sandberg D., Wahde M., Philip P., Kronberg P. (2010). Reaction of sleepiness indicators to partial sleep deprivation, time of day and time on task in a driving simulator - the DROWSI project. J. Sleep Res..

[51] Wang X., Xu C. (2016). Driver drowsiness detection based on non-intrusive metrics considering individual specifics. Accid. Anal. Prev..

[52] Vinckenbosch F.R.J., Vermeeren A., Verster J.C., Ramaekers J.G., Vuurman E.F. (2020). Validating lane drifts as a predictive measure of drug or sleepiness induced driving impairment. Psychopharmacology (Berl).

[53] Thiffault P., Bergeron J. (2003). Monotony of road environment and driver fatigue: a simulator study. Accid. Anal. Prev..

[54] Mallis M.M., Dinges D.F. (2004). Handb. Hum. Factors Ergon. Methods.

[55] Sahayadhas A., Sundaraj K., Murugappan M. (2012). Detecting driver drowsiness based on sensors: a review. Sensors.

[56] Wierwille W.W., Ellsworth L.A. (1994). Evaluation of driver drowsiness by trained raters. Accid. Anal. Prev..

[57] McDonald A.D., Lee J.D., Schwarz C., Brown T.L. (2018). A contextual and temporal algorithm for driver drowsiness detection. Accid. Anal. Prev..

[58] Van Loon R.J., Brouwer R.F.T., Martens M.H. (2015). Drowsy drivers' under-performance in lateral control: how much is too much? Using an integrated measure of lateral control to quantify safe lateral driving. Accid. Anal. Prev..

[59] Singmann H., Kellen D. (2019). An introduction to mixed models for experimental psychology. New Methods Cogn. Psychol.

[60] Satterthwaite F.E. (1946). An approximate distribution of estimates of variance components. Biometrics Bull..

[61] Kuznetsova A., Brockhoff P.B., Christensen R.H.B. (2017). lmerTest package: tests in linear mixed effects models. J. Stat. Software.

[62] Schielzeth H., Dingemanse N.J., Nakagawa S., Westneat D.F., Allegue H., Teplitsky C., Réale D., Dochtermann N.A., Garamszegi L.Z., Araya-Ajoy Y.G. (2020). Robustness of linear mixed-effects models to violations of distributional assumptions. Methods Ecol. Evol..

[63] Box G.E.P., Cox D.R. (1964). An analysis of transformations. J. R. Stat. Soc. Ser. B.

[64] McCullagh P. (1980). Regression models for ordinal data. J. R. Stat. Soc. Ser. B.

[65] Bürkner P.C., Vuorre M. (2019). Ordinal regression models in psychology: a tutorial. Adv. Methods Pract. Psychol. Sci..

[66] Bates D., Mächler M., Bolker B.M., Walker S.C. (2015). Fitting linear mixed-effects models using lme4. J. Stat. Software.

[67] Christensen R.H.B. (2018).

[68] Lenth R., Singmann H., Love J., Buerkner P., Herve M. (2020). emmeans: estimated marginal means. R package version 1.4. 4. Am. Statistician.

[69] Brookhuis K.A., De Waard D., Fairclough S.H. (2003). Criteria for driver impairment. Ergonomics.

[70] Gastaldi M., Rossi R., Hadas Y., Fasan D., Keren N., Mulatti C. (2016).

[71] van der Sluiszen N.N.J.J.M., Vermeeren A., Jongen S., Theunissen E.L., van Oers A.C.M., Van Leeuwen C.J., Maret A., Desforges C., Delarue A., Ramaekers J.G. (2016). On-the-road driving performance after use of the antihistamines mequitazine and l-mequitazine, alone and with alcohol. Psychopharmacology (Berl)..

[72] Kuypers K.P.C., Samyn N., Ramaekers J.G. (2006). MDMA and alcohol effects, combined and alone, on objective and subjective measures of actual driving performance and psychomotor function. Psychopharmacology (Berl)..

[73] Leufkens T.R.M., Lund J.S., Vermeeren A. (2009). Highway driving performance and cognitive functioning the morning after bedtime and middle-of-the-night use of gaboxadol, zopiclone and zolpidem. J. Sleep Res..

[74] Mets M.A.J., De Vries J.M., De Senerpont Domis L.M., Volkerts E.R., Olivier B., Verster J.C. (2011). Next-day effects of ramelteon (8 mg), zopiclone (7.5 mg), and placebo on highway driving performance, memory functioning, psychomotor performance, and mood in healthy adult subjects. Sleep.

[75] Cellini N., Bruno G., Orsini F., Vidotto G., Gastaldi M., Rossi R., Tagliabue M. (2023). The effect of partial sleep deprivation and time-on-task on young drivers' subjective and objective sleepiness. Int. J. Environ. Res. Publ. Health.

[76] Kumar Yadav A., Velaga N.R. (2021). A comprehensive systematic review of the laboratory-based research investigating the influence of alcohol on driving behaviour. Transport. Res. F Traffic Psychol. Behav..

[77] Helland A., Jenssen G.D., Lervåg L.-E., Westin A.A., Moen T., Sakshaug K., Lydersen S., Mørland J., Slørdal L. (2013). Comparison of driving simulator performance with real driving after alcohol intake: a randomised, single blind, placebo-controlled, cross-over trial. Accid. Anal. Prev..

[78] Kenntner-Mabiala R., Kaussner Y., Jagiellowicz-Kaufmann M., Hoffmann S., Krüger H.P. (2015). Driving performance under alcohol in simulated representative driving tasks: an alcohol calibration study for impairments related to medicinal drugs. J. Clin. Psychopharmacol..

[79] Zamarripa C.A., Novak M.D., Weerts E.M., Vandrey R., Spindle T.R. (2022). The effects of oral and vaporized cannabis alone, and in combination with alcohol, on driving performance using the STISIM driving simulator: a two-part, double-blind, double-dummy, placebo-controlled, randomized crossover clinical laboratory protocol. Front. Pharmacol..

[80] Abe T. (2023). PERCLOS-based technologies for detecting drowsiness: current evidence and future directions. SLEEP Adv.

[81] Martínez-Pérez V., Palmero L.B., Campoy G., Fuentes L.J. (2020). The role of chronotype in the interaction between the alerting and the executive control networks. Sci. Rep..

[82] Wagner U., Gais S., Haider H., Verleger R., Born J. (2004). Sleep inspires insight. Nature.

[83] Weast R. (2018). Temporal factors in motor-vehicle crash deaths: ten years later. J. Saf. Res..

[84] Radun I., Radun J.E. (2006). Seasonal variation of falling asleep while driving: an examination of fatal road accidents. Chronobiol. Int..

